# OVAT Analysis and Response Surface Methodology Based on Nutrient Sources for Optimization of Pigment Production in the Marine-Derived Fungus *Talaromyces albobiverticillius* 30548 Submerged Fermentation

**DOI:** 10.3390/md19050248

**Published:** 2021-04-27

**Authors:** Mekala Venkatachalam, Alain Shum-Chéong-Sing, Yanis Caro, Laurent Dufossé, Mireille Fouillaud

**Affiliations:** 1 Laboratoire de Chimie et Biotechnologie des Produits Naturels—ChemBioPro, Université de la Réunion, 15 Avenue René Cassin, CS 92003, CEDEX 9, F-97744 Saint-Denis, Ile de la Réunion, France; mekalavenkat@gmail.com (M.V.); alain.shum@univ-reunion.fr (A.S.-C.-S.); yanis.caro@univ-reunion.fr (Y.C.); laurent.dufosse@univ-reunion.fr (L.D.); 2 Ecole Supérieure d’Ingénieurs Réunion Océan Indien—ESIROI, 2 Rue Joseph Wetzell, F-97490 Sainte-Clotilde, Ile de la Réunion, France

**Keywords:** fungi, *Talaromyces albobiverticillius* 30548, pigments, optimization, biomass, one-variable-at-a-time, OVAT, central composite design, CCD, RSM

## Abstract

Pigment production from filamentous fungi is gaining interest due to the diversity of fungal species, the variety of compounds synthesized, and the possibility of controlled massive productions. The *Talaromyces* species produce a large panel of metabolites, including *Monascus-like* azaphilone pigments, with potential use as natural colorants in industrial applications. Optimizing pigment production from fungal strains grown on different carbon and nitrogen sources, using statistical methods, is widespread nowadays. The present work is the first in an attempt to optimize pigments production in a culture of the marine-derived *T. albobiverticillius* 30548, under the influence of several nutrients sources. Nutrient combinations were screened through the one-variable-at-a-time (OVAT) analysis. Sucrose combined with yeast extract provided a maximum yield of orange pigments (OPY) and red pigments (RPY) (respectively, 1.39 g/L quinizarin equivalent and 2.44 g/L Red Yeast pigment equivalent), as well as higher dry biomass (DBW) (6.60 g/L). Significant medium components (yeast extract, K_2_HPO_4_ and MgSO_4_·7H_2_O) were also identified from one-variable-at-a-time (OVAT) analysis for pigment and biomass production. A five-level central composite design (CCD) and a response surface methodology (RSM) were applied to evaluate the optimal concentrations and interactive effects between selected nutrients. The experimental results were well fitted with the chosen statistical model. The predicted maximum response for OPY (1.43 g/L), RPY (2.59 g/L), and DBW (15.98 g/L) were obtained at 3 g/L yeast extract, 1 g/L K_2_HPO_4_, and 0.2 g/L MgSO_4_·7H_2_O. Such optimization is of great significance for the selection of key nutrients and their concentrations in order to increase the pigment production at a pilot or industrial scale.

## 1. Introduction

A number of food additives perform certain technological functions in improving food quality, yet, color is often the first element noticed in the appearance of a food product. Synthetic or artificial colors remain the most popular when compared to natural colorants, as they are brighter, more uniform, better characterized, and of higher tinctorial strength. Moreover, they encompass a wider range of hues, and, most of all, are less expensive [1,2]. Although some artificial colorants were approved for food use, organizations, such as the U.S. Food and Drug Administration (FDA) and the European Food Safety Authority (EFSA) instituted warnings on consumption, and recommended safe dosages in foods, cosmetics, and drugs [3,4,5]. Nonetheless, many colors were banned due to the potential disadvantages for consumers, as they may be linked to hyperactivity disorders, cancer, allergies, and toxicological problems [6].

Thus, there has been increasing interest in natural pigment producing agents as a replacement for synthetic dyes. Among natural sources, microorganisms are receiving increasing interest, owing to their easy cultivation, potential massive production in cheap nutrient media, and high pigment stability [7,8,9]. Fungal species are extensively studied for their potential as pigment sources, as they produce an astonishing variety of compounds, including carotenoids, melanins, flavins, phenazines, quinones, monascins, violacein, and indigo, exhibiting a wide range of colors [10,11,12]. Fungal pigments are considered as secondary metabolites, whose production usually begins after active cell growth. Furthermore, pigments are often produced by fungal mycelia to survive in adverse situations (e.g., insufficient nutrient supply, unfavorable physicochemical parameters, or antagonistic microbial interactions) [13,14,15]. Due to the uncertainty of the intrinsic biological role of these compounds, and the timeline of their appearance in cell cycles, a new trend involves qualifying these metabolites as specialized instead of secondary [16]. Moreover, fungal pigments are reported to demonstrate bioactive properties that are of considerable interest for new drug development in pharmaceuticals [17,18].

Biosynthetically, most pigments produced by fungi are polyketide-based and involve complex pathways catalyzed by frequent types of polyketide synthases (PKs) [19,20]. The biosynthesis of azaphilones uses both the polyketide pathway and the fatty acid synthesis pathway. Biosynthetic pathways are suggested for the following azaphilones: monascorubrin and monascoflavin [21], mitorubrin, and rubropunctatin [22] or citrinin [23,24]. However, some researchers are of the opinion that biosynthetic mechanisms of these pigments are poorly understood, including the extensively studied *Monascus* pigments [25].

For the purpose of industrial production, the physicochemical conditions for the production of pigments are of great concern. In many cases, microbial pigments are better produced in liquid submerged fermentation (SmF) compared to solid substrate fermentation (SSF). Indeed, SmF allows homogenous growth of cells when supported with appropriate agitation under controlled conditions [2,26]. Various ascomycetes fungi, including *Drechslera* sp. [27,28], *Fusarium* sp. [29,30,31], *Monascus* sp. [32,33,34,35], *Paecilomyces* sp. [36,37], *Penicillium* sp. [38,39,40,41,42,43], *Talaromyces* sp. [44,45] or *Cordyceps unilateralis* [46], *Curvularia lunata, Herpotrichia rhodosticta* [47] were described to produce pigments by fermentative technology. In particular, for *Monascus spp.,* studies have revealed that numerous environmental factors regulate the ability of pigment production, particularly the medium pH and a nitrogen source [48,49,50,51,52,53,54,55].

Optimization of medium components is a tedious process due to the involvement of numerous factors influencing fermentation. Thus, the formulation of culture media containing complex nutrients is generally preferred to reduce the production cost and give maximum product yield [56,57,58]. In the classical optimization strategy, only one factor or variable is varied at a time (OVAT: one-variable-at–a–time analysis), while keeping other variables constant. Because of its ease and convenience, the OVAT analysis has been the most preferred choice among researchers for designing the media composition. However, this methodology is mainly used in the initial stages in diverse research fields [59], especially during the medium formulation for the production of new metabolites, or known compounds from a new source. Indeed, as soon as the number of parameters increases, the evaluation of their effects and interactions become more complex.

After determining the critical factors through OVAT, the next step is to optimize the actual values of these process factors. The statistical design of experiments is a powerful approach for media optimization, and offers a systematic way of simultaneously evaluating multiple parameter effects and analyzing the resulting process outputs. To achieve this purpose, a central composite response surface methodology (RSM) can be used. This empirical technique makes it possible to evaluate the relationship between independent variables, and to predict the selected responses [60,61]. For *Monascus* spp., experiments were continuously made to select better carbon and nitrogen sources, for a higher production of pigments [62,63,64,65,66]. With this in mind, a study was undertaken to focus on simple and the most suitable nutrient medium for pigment production using the marine-derived fungal strain *T. albobiverticillius* 30548.

## 2. Materials and Methods

### 2.1. Microorganism and Cultivation

The studied strain, *T. albobiverticillius* 30548, was isolated from a marine sediment sample collected around Réunion Island, Indian Ocean [67]. From the taxonomic identification (Westerdijk Fungal Biodiversity Institute, Utrecht, The Netherlands), the fungus was identified as *T. albobiverticillius* and named as *T. albobiverticillius* 30548 (GenBank accession number MK937814). The fungus was grown on potato dextrose agar (PDA, Difco, France) at 25 °C for 7 days and maintained at 4 °C, as well as sub-cultured at regular intervals for the experiments.

### 2.2. Primary Screening of Medium Components by OVAT Analysis

In previous studies, the major influencing factors (pH, temperature, agitation speed, and fermentation time) for fungal growth and pigment production from *T. albobiverticillius* 30548 were screened and optimized by culturing the strain in potato dextrose broth alone (PDB, BD Difco, Franklin Lakes, NJ, USA) [68]. Then, to determine the most appropriate nutrient sources for maximum pigments production, different carbon and nitrogen sources were chosen for an OVAT study (one-variable-at-a-time analysis). The concentration ranges of the factors were chosen based on the existing literature data and available reports [69,70]. Preliminary experiments were carried out using five different carbon sources (glucose, sucrose, fructose, soluble starch, and malt extract); 4 nitrogen sources (sodium nitrate, peptone, tryptone and yeast extract), and different inorganic salts (K_2_HPO_4_, MgSO_4_·7H_2_O, FeSO_4_·7H_2_O, KCl), were used to evaluate their suitability for fungal growth and pigment production by the fungal strain. All were purchased from Sigma-Aldrich, Darmstadt, Germany. The chemical composition of the medium was fixed as follows (g/L): 15, carbon source; 3, nitrogen source; 1, K_2_HPO_4_; 0.2, MgSO_4_·7H_2_O; 0.2, FeSO_4_·7H_2_O; 0.25, KCl. The working stocks of culture media components, such as carbon source, nitrogen source, and trace elements were prepared and sterilized separately (121 °C, at 15 psi for 15 min). Upon cooling to room temperature, the sterile components were mixed aseptically at appropriate proportions.

### 2.3. Submerged Fermentations

A small loop of fungal mycelia grown on PDA was transferred into 80 mL of sterile PDB culture medium. The flasks were incubated at 24 °C for 48 h with 150 rpm of agitation in an orbital rotary shaker (Multitron Pro, Infors HT, Bottmingen, Switzerland), and considered as pre-inoculum. After 48 h of fermentation, the culture broth was allowed to centrifuge at 8000 rpm for 6 min at room temperature (Sigma 4K15, Merck, Darmstadt, Germany), to separate fungal mycelia from culture filtrate. The harvested mycelium (100 mg) was transferred as seed inoculum into a 200 mL Erlenmeyer flask containing 80 mL of formulated sterile fermentation medium. The flasks were then incubated at 24 °C under 200 rpm for 10 days. All experiments were performed as triplicates and pigment production was monitored on an everyday basis.

### 2.4. Quantification of Pigments

Throughout the period of cultivation, and for each condition, 5 mL of culture was withdrawn from the flasks to measure the optical density of the fermentation liquid. The sample was centrifuged at 8000 rpm (Sigma 4K15, Merck, Darmstadt, Germany), for 6 min at room temperature to separate the supernatant from the mycelium. The color-rich supernatant, containing the extracellular pigments, was quantified using a UV–Visible spectrophotometer (UV–Vis spectrophotometer UV-1800, Shimadzu, Kyoto, Japan). As the fungus produces a mixture of colored compounds [71], the absorbance of the pigmented solution was measured at 470 and 500 nm, representing the regions for orange and red colors, respectively [72,73]. Pigment yields were observed as OD units at their maximum wavelength of absorbance and the values were converted into g/L equivalents (g/L equivalent) using selected reference standards. Quinizarin (orange hue) (Sigma-Aldrich, Darmstadt, Germany) and Red Yeast rice pigments (RYrp, red hue) (Wuhan Jiacheng Biotechnology Co. Ltd., Wuhan, China) were used as reference standards, since the colors and absorbance profiles of the major pigments produced by *T. albobiverticillius* 30548 were similar to those of these two compounds (see details in [67]). OD values at 470 (orange) and 500 nm (red) were interpolated using, respectively, the standard curve equations of quinizarin and RYrp, to obtain pigment concentrations in terms of g/L equivalents. The concentrations of orange and red pigments in g/L equivalent were consequently calculated using the below represented formula (Equations (1) and (2)): 

Orange pigment concentration:

(1) $$C_{\left(g/L\;equiv.\;\;quinizarin\right)}=\frac{Abs_{470}-0.1102}{0.0148}\times 0.001$$

Red pigment concentration:

(2) $$C_{\left(g/L\;equiv.\;\;RYrp\right)}=\frac{Abs_{500}-0.0968}{0.0069}\times 0.001$$

### 2.5. Dry Biomass Concentration

The fungal biomass was estimated as dry weight by separating the fermentation broth and mycelia using 48 µm nylon mesh filter (SEFAR, Nitex, Heiden, Switzerland). The filters containing the collected biomass were dried in a hot air oven (SNB 100, Memmert, Schwabach, Germany) at 105 °C for 17 h. The dried filters were left to cool in a desiccator for 30 m until they reached the room temperature, and were then weighed. The concentration of dry biomass in the culture broth, denominated as DBW, is calculated from the following equation (Equation (3)):

(3) $${\left[DBW\right]}_{\left(g/L\;\right)}=\frac{\left(W2-W1\right)}{V}\times 0.001$$

where, *W*_2_ is the weight of the filter with biomass obtained after drying (*g*), *W*_1_ is the weight of the corresponding empty filter (*g*), and V is the volume of sample (*L*).

### 2.6. Optimization Using Central Composite Design (CCD) and Response Surface Modeling (RSM)

To determine the optimum concentration of the most effective factors identified by the OVAT (one-variable-at-a-time) analysis, and to study their interactions, a response surface methodology (RSM) approach, in terms of a central composite design (CCD), was applied. Design-Expert^®^ Software (Version 9.0.3.1, Stat-Ease Inc., Minneapolis, MN, USA) was used in this study to construct the experimental design and statistically analyze the experimental data. Based on the OVAT results, the basic chemical composition of the medium was fixed as follows (g/L): 15, sucrose; 0.2, FeSO_4_·7H_2_O; 0.25, KCl added to different concentrations of yeast extract, KH_2_PO_4_, and MgSO_4_·7H_2_O. Then, the pre-defined key variables were denoted as follows: yeast extract [X_1_], KH_2_PO_4_ [X_2_], and MgSO_4_·7H_2_O [X_3_]. Each variable in the design was studied at five different levels, with all variables taken at a central coded value of zero (Table 1).

A factorial experimental design, with an axial point (α = 1.68) and six replicates at the center point, was employed, with a total of 20 experiments (Table 2). The responses Y1, Y2, Y3, refer to the orange pigment yield (OPY), red pigment yield (RPY), and dry biomass concentration (DBW), respectively. A multiple regression analysis of the data was carried out to draw out an empirical model linking the response measured (Y) to the independent variables (X1, X2, X3). The following second-order polynomial equation describes the relationship between the independent variables and allows the calculation of the expected responses (Equation (4)):

(4) $$Y=\beta_{0}+\overset{k}{\underset{j=1}{\Sigma }}\beta_{j}x_{j}+\overset{k}{\underset{j=1}{\Sigma }}\beta_{jj}x_{j}^{2}+\underset{i}{\Sigma }\overset{k}{\underset{<j=2}{\Sigma }}\beta_{ij}x_{i}x_{j}+e_{i}$$

where Y is the response; *x_i_* and *x_j_* are variables (*i and j range from* 1 to *k*); *β_0_* is the model intercept coefficient; *β_j_, β_jj_*, and *β_ij_* are interaction coefficients of the linear, quadratic and second-order terms, respectively; *k* is the number of independent parameters *(k* = 3 in this study); and *e_i_* is the error [74,75].

### 2.7. Statistical Analysis and Data Validation

Design-Expert^®^ Software (Version 9.0.3.1, Stat-Ease Inc., Minneapolis, MN, USA) was used to obtain the response surface models for the CCD. For the data analysis, ANOVA through Fisher’s test was applied to evaluate the effect of independent variables on the responses. The significant results were identified by a *p*-value < 0.05. Multiple correlation coefficient (R^2^) and adjusted R^2^ (R^2^ adj.) were used as quality indicators to evaluate the fitness of the second order polynomial equation. Contour and three-dimensional surface plots (RSM) were employed to demonstrate the relationship and interaction between the coded variables and the responses. The optimal points were determined by solving the equation derived from the final quadratic model and grid search in RSM plots. Each experiment represents the mean of three independent experiments, and the results were presented as mean ± SD.

### 2.8. C/N Ratio Calculation and Means Comparison

Global C/N ratios for each nutrient combination tested in CCD (see Table 2) were calculated using the data available in Thompson et al. [76]. The carbon content of yeast extract was estimated at 40% and its nitrogen content at 40%. The carbon content of sucrose was calculated as 42.11%. For mean comparisons, ANOVA was realized using XLStat (v2021.1, Addinsoft, Paris, France). For the multiple comparison test, REGWQ (Ryan, Einot, Gabriel, Welsh Studentized Range Q test) procedure was conducted under 5% error.

## 3. Results

### 3.1. Primary Screening of Media Components Using OVAT Analysis

Among the tested carbon and nitrogen sources, some showed positive and some showed minimum effects on pigment production. The experimental combinations used in OVAT analysis are detailed in Table 1.

**Figure 1:**
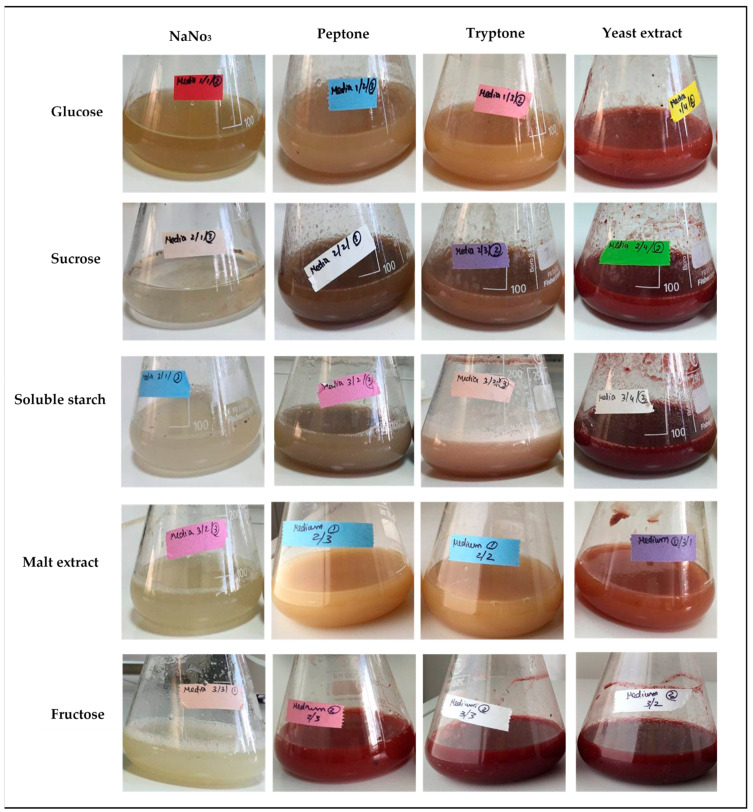
Images of pigment production by *T. albobiverticillius* 30548 in various culture media with combinations of: carbon source 15 g/L, nitrogen source 3 g/L, K_2_HPO_4_ 1 g/L, MgSO_4_·7H_2_O 0.2 g/L, FeSO_4_·7H_2_O, 0.25 g/L and KCl 0.1 g/L, after 8 days of cultivation (temperature 24 °C, 150 rpm agitation speed, and initial pH = 6.4).

Figure 1 presents the exemplary results of the pigment production in 80 mL of fermentation media with the different carbon and nitrogen sources. The highest pigment production with red hue was identified for the media containing yeast extract, whatever the carbon source was. Nevertheless, fructose combination with peptone or tryptone also exhibited significant red shades.

**Figure 2:**
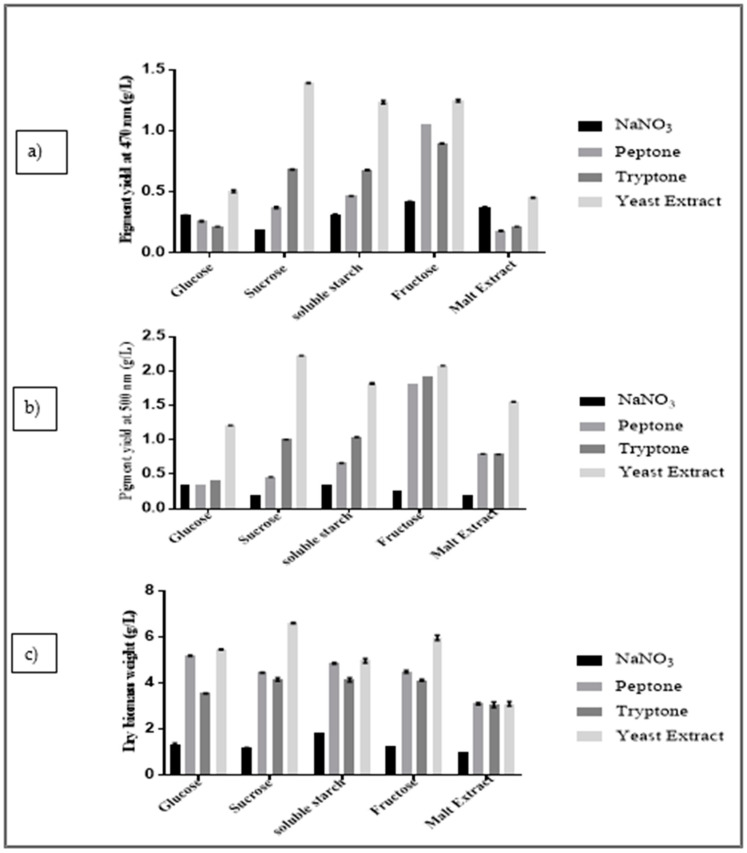
The main effects of combined carbon and nitrogen sources on (**a**) OPY (orange pigment yield at 470 nm in terms of g/L quinizarin equivalent), (**b**) RPY (red pigment yield at 500 nm in terms of g/L red yeast rice pigment equivalent), (**c**) DBW (g/L dry biomass concentration) for *T. albobiverticillius* 30548. Data plotted are means ± SD of three replicate growths per culture medium.

The results of the pigment quantification presented in Figure 2a–c indicate that, when sucrose and yeast extract were used as combined sources (15 g sucrose and 3 g of yeast extract with essential trace elements for fungal growth), it provided a maximum yield of orange pigments (OPY = 1.39 g/L quinizarin equivalent) and red pigments (RPY = 2.44 g/L RYrp equivalent), and higher dry biomass weight (DBW = 6.60 g/L).

Fructose with yeast extract was the second-best combination (OPY = 1.24 g/L quinizarin equivalent; RPY = 2.07 g/L RYrp equivalent; DBW = 5.96 g/L), immediately followed by its association with peptone or tryptone.

Peptone and tryptone individually combined with any of the carbon sources provided nearby yields, always lower than yeast extract.

Sodium nitrate used as an inorganic nitrogen source provided very low yields among all of the nitrogen sources tested.

In the context of the above observations, sucrose and yeast extract were shown to improve the yield of responses.

### 3.2. Optimization of Components Concentrations by Central Composite Design (CDD) and Response Surface Methodology (RSM)

- Development of second order polynomial models:

In this study, using Design-Expert^®^ Software (Version 9.0.3.1), CCD with three factors at five levels was employed to investigate the influence of process variables. The concentrations of nutrients (carbon source, nitrogen source, and salts) were screened for maximum yields of pigments (OPY for orange and RPY for red), and fungal biomass (DBW) from *T. albobiverticillius* 30548 in SmF. Twenty experiments were carried out with different combinations of factors, their levels and the responses are tabulated in Table 2.

An empirical relationship expressed by a second-order polynomial equation with interaction terms was fitted between the experimental results obtained on the basis of CCD and the input variables. The observed experimental results in each run were subjected to multiple regression analyses to calculate the regression coefficients of the model. Calculated regression coefficients were substituted in Equation (4) to obtain a model for orange pigment yield OPY Equation (5), red pigment yield RPY Equation (6), and dry biomass concentration DBW Equation (7). These equations assert the relationship between the process factors and the responses. After neglecting the insignificant factors, this can be presented in terms of coded variables as:

**OPY** = −0.52 + 0.89 X_1_ + 0.63 X_2_ + 3.30 X_3_ + 0.02 X_1 × 2_ − 0.01 X_1_X_3_ − 0.37 X_2_X_3_ − 0.16 X_1_^2^ − 0.34 X_2_^2^ − 5.94 X_3_^2^, (5)

**RPY** = −4.07 + 2.64 X_1_ + 3.41 X_2_ + 8.80 X_3_ − 0.007 X_1_X_2_ − 0.012 X_1_X_3_ + 0.12 X_2_X_3_ − 0.43 X_1_^2^ −1.70 X_2_^2^ −21.44 X_3_^2^, (6)

**DBW** = −22.09 + 17.32 X_1_ + 11.90 X_2_ + 44.53 X_3_ − 0.02 X_1_X_2_ + 2.81 X_1_X_3_ − 0.88 X_2_X_3_ – 3.10 X_1_^2^ − 5.74 X_2_^2^ − 99.45 X_3_^2^, (7)

where, X_1_ represents yeast extract, X_2_ is KH_2_PO_4_ and X_3_ is MgSO_4_·7H_2_O.

### 3.3. Analytical Validation

A multiple regression analysis using the least square method was carried out to test the adequacy and fitness of the models. An ANOVA was also applied to check its significance. The results are presented in Table 3, Table 4 and Table 5. The significance of each variable was thus evaluated through an analysis of variance followed by Fisher’s statistical test (F-test). This indicated that most of the variations in the response can be explained by the developed regression equations, according to the high F-values: 51.09 for orange pigment yield (OPY) measured at 470 nm, 55.81 for red pigment yield (RPY) measured at 500 nm and 23.77 for dry biomass weight (DBW). The associated p-values, lower than 0.05, indicated that F was large enough and that the developed model and the terms were statistically significant. Indeed, the p-values, lower than 0.0001 for all the responses (OPY, RPY, DBW), demonstrated the precision and the accuracy of the constructed models.

The adequacy and accuracy of the developed models were controlled using the determination coefficient (R^2^), adjusted R^2^ (R^2^_adj._), predicted R^2^ (R^2^_pred._), and coefficient of variation (CV%). R^2^ represented the proportion of total variation in the responses predicted by the models. In our work, ANOVA demonstrated that the determination coefficient (R^2^) was 0.9978 for OPY, 0.9998 for RPY, and 0.9553 for DBW, respectively. According to these values of R^2^, the fit of the quadratic model to the experimental data is correct. This indicates that only 0.22, 0.02 and 4.47% of the deviations for OPY, RPY, and DBW, respectively remain unexplained [77].

The adjusted determination coefficient (R^2^_adj._) allows the correction of the R^2^ value, taking into account the sample size and the number of terms in the model. If needless variables are enclosed in the model, the adjusted R^2^ (R^2^_adj._) will diminish and will be lower than or equal to R^2^. The values of R^2^_adj._ calculated here (0.9959 for OPY at 470 nm, 0.9996 for RPY at 500 nm, and 0.9151 for DBW) were also high and notably close to the R^2^ values, particularly for pigment production. This indicated an appropriate prediction of the model. The difference observed between these two parameters for DBW modeling, may indicate some of the selected variables show a weaker link with the biomass production. R^2^_pred._ is a measure of how good the model predicts a response value. In our case, the R^2^_pred_ (0.9859 for OPY, 0.9988 for RPY, and 0.8209 for DBW) are in reasonable agreement with the R^2^_adj_.

With the coefficient of variation (CV%) indicating the relative dispersion of the experimental points from the predictions of the second-order polynomial models, it is possible to determine the degree of precision and reliability of the conducted experiments. A minimum value of CV% (0.88% for OPY, 0.55% for RPY, and 8.85% for DBW) interprets the low deviation between the experimental and predicted value and indicates a good degree of precision. Signal to noise ratio is referred to as adequate precision (Adeq. Pre.), and a value greater than 4 is desirable [78]. In this study, the adequate precision was less than 15 for the three responses, and proved the model can be used to navigate the design space.

Table 3, Table 4 and Table 5 interpret that the significance of the developed model is sharp and can adequately explain the real relationship among the independent variables and the responses, in the chosen range. The model chosen satisfactorily explains the square of parameters (*p* < 0.05 for X_1_^2^, X_2_^2^, X_3_^2^) and interaction effects (*p* < 0.05 for X_1_X_2_, X_1_X_3_, X_2_X_3_) of selected medium components on pigment production by *T. albobiverticillius 30548* in shake flasks SmF.

### 3.4. Effect of Process Variables and Responses (RSM)

**Figure 3:**
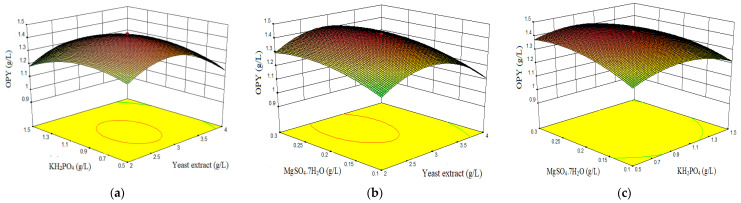
3D surface plots showing the interactive effects of factors on orange pigment yield (OPY). (**a**) Effect of KH_2_PO_4_ and yeast extract. (**b**) Effect of MgSO_4_·7H_2_O and yeast extract. (**c**) Effect of MgSO_4_·7H_2_O and KH_2_PO_4_.

**Figure 4:**
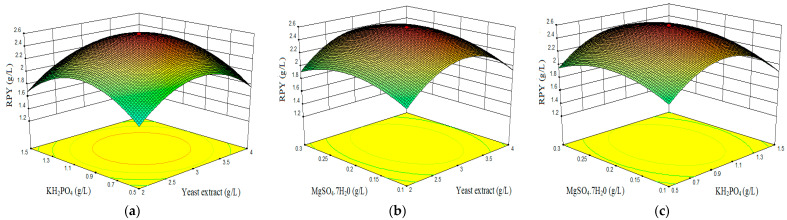
3D surface plots showing the interactive effects of factors on red pigment yield (RPY). (**a**) Effect of KH_2_PO_4_ and yeast extract. (**b**) Effect of MgSO_4_·7H_2_O and yeast extract. (**c**) Effect of MgSO_4_·7H_2_O and KH_2_PO_4_.

**Figure 5:**
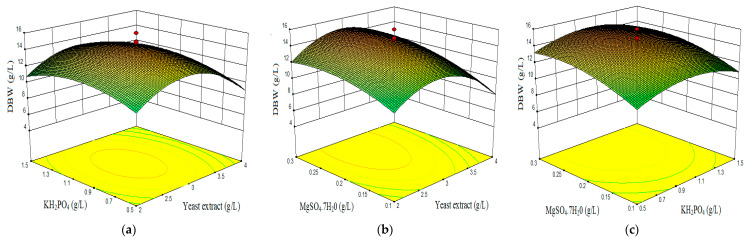
3D surface plots showing the interactive effects of factors on dry biomass weight (DBW). (**a**) Effect of KH_2_PO_4_ and yeast extract. (**b**) Effect of MgSO_4_·7H_2_O and yeast extract. (**c**) Effect of MgSO_4_·7H_2_O and KH_2_PO_4_.

From the developed model Equations (5)–(7), three-dimensional response graphs were plotted to visualize the relationship between factors and responses. Response surface curves for the variation in pigment production (orange and red) and dry biomass weight were constructed, depicted in Figure 3, Figure 4 and Figure 5. In each set, responses were plotted on Z axis against two factors, while maintaining the last factor at constant at its middle level [79,80].

- Combined effect of yeast extract, KH_2_PO_4_, and MgSO_4_·7H_2_O on orange pigment yield (OPY).

Figure 3a depicts the production of orange pigments with respect to yeast extract versus KH_2_PO_4_. From the interaction response, orange pigment yield increased with increasing yeast extract and KH_2_PO_4_ concentrations up to 2.9 g/L and 0.9 g/L, respectively.

Figure 3b represents the interaction effect of yeast extract and MgSO_4_·7H_2_O on the production of orange pigment. With an increase in yeast extract (2.0–2.9 g/L) and MgSO_4_·7H_2_O (0.1–0.2 g/L) concentration, OPY increased. Thereafter, an increase in MgSO_4_·7H_2_O concentration up to 0.3% (*w/v*) resulted in decreased orange pigment production.

Figure 3c reveals the orange pigment production in relation with variations of KH_2_PO_4_ and MgSO_4_·7H_2_O. The maximum range was found to be 0.2 g/L MgSO_4_·7H_2_O and 0.9 g/L KH_2_PO_4_. However, the response curve did not show curvature, rather it was flattened. This suggests a clear demand for yeast extract rather than the influence of trace elements (MgSO_4_·7H_2_O and KH_2_PO_4_) in relation with the production of orange pigment. Thus, the maximum OPY was 1.43 g/L quinizarin equivalent, within the tested nutrients concentrations.

- Combined effect of yeast extract, KH_2_PO_4_, and MgSO_4_·7H_2_O on red pigment yield (RPY).

Figure 4a–c illustrate the interaction effect of the three individual factors on red pigment production. In all the three graphs, (interaction of yeast extract with KH_2_PO_4_ (Figure 4a), yeast extract versus MgSO_4_·7H_2_O (Figure 4b), and KH_2_PO_4_ versus MgSO_4_·7H_2_O (Figure 4c)), there was an increased red pigment production in the range of 2.9 g/L yeast extract, 0.2 g/L MgSO_4_·7H_2_O, and 0.9 g/L KH_2_PO_4_. In this concentration range, the red pigment production was maximum with a value of 2.59 g/L of RYrp equivalent.

- Combined effect of yeast extract, KH_2_PO_4_, and MgSO_4_·7H_2_O on dry biomass weight (DBW).

Figure 5a,b exemplify the interaction effect of yeast extract with KH_2_PO_4_ (Figure 5a) or with MgSO_4_·7H_2_O (Figure 5b) on dry biomass weight. A clear increase of biomass until 2.9 g/L yeast extract is observed, and then DBW starts to decrease in amount. The influence of KH_2_PO_4_ and MgSO_4_·7H_2_O appears not very high on biomass production as it is shown on graphs. Moreover, Figure 5c depicts the interaction of KH_2_PO_4_ combined with MgSO_4_·7H_2_O. Increasing the phosphate to 0.9 g/L and sulfate to 0.2 g/L concentrations just slightly increased the biomass content toward the end (15.98 g/L).

At the chosen concentration ranges (0.5–1.5 g/L for KH_2_PO_4_, 0.1–0.3 g/L for MgSO_4_·7H_2_O, and 2–4 g/L for yeast extract) the variations of yeast extract concentration demonstrate a higher impact on red and orange pigments production as well as on DBW, compared to KH_2_PO_4_ and MgSO_4_·7H_2_O effects. The highest influence of these two compounds concentrations is observed on RPY, and the KH_2_PO_4_ level seems more influential.

### 3.5. Model Validation

The validation was carried out in shake flasks under optimum conditions of the medium components predicted by the model. From the model, the predicted maximum response of OPY (1.43 g/L), RPY (2.59 g/L), and DBW (15.98 g/L) were obtained at the optimal level of medium variables: 3 g/L yeast extract, 1 g/L K_2_HPO_4_, and 0.2 g/L MgSO_4_·7H_2_O. To verify the accuracy of the model, a validation experiment was carried out at the predicted optimal concentrations of these components. In the validation experiment, maximum orange and red pigment yields of 1.45 g/L (OPY) and 2.57 g/L (RPY) were obtained. The amount of dry biomass weight produced under optimized conditions was 16.11 g/L showing a good coincidence with the predicted value. The accuracy of the fitted model was found to be 0.9859. Results of validation explained that the predicted model for OPY, RPY, and DBW was well fitted with the experimental results.

### 3.6. C/N Ration Influence in CCD Experiments

Based on Table 2 data and the corresponding RSM in the chosen concentration range, the influence of variable X1 (KH_2_PO_4_) and X3 (MgSO_4_·7H_2_O) on the responses are weak. Therefore, a preliminary approach of the influence of C/N ratio on cell growth and pigments production can be suggested (Table 6).

**Figure 6:**
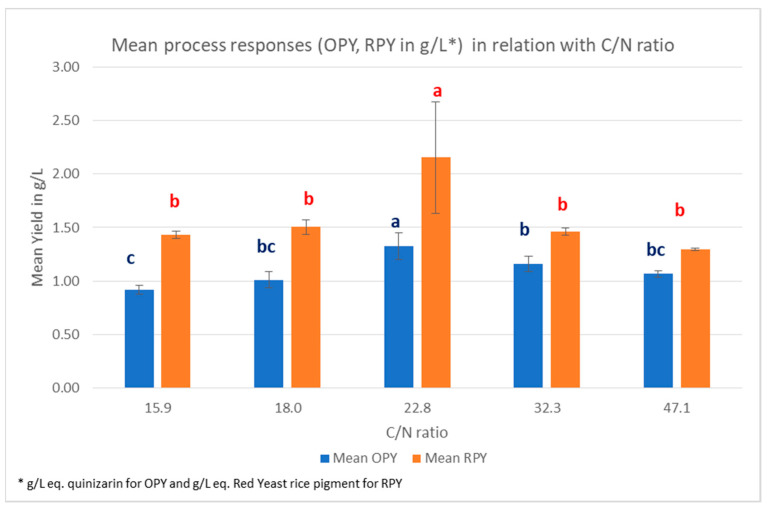
Mean responses for OPY (orange pigment yield at 470 nm) and RPY (red pigment yield at 500 nm) in relation with C/N ratio in the culture medium for *T. albobiverticillius* SmF. The significance of differences between the values are illustrated with letters. Different letters (in the same color) indicate significant differences between the experimental values.

**Figure 7:**
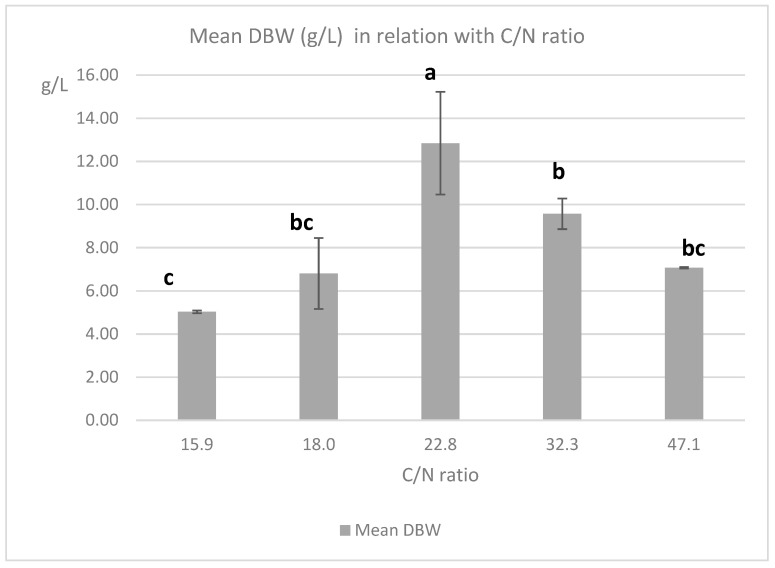
Mean responses for DBW (dry biomass concentration) in relation with C/N ratio in the culture medium for *T. albobiverticillius* SmF. The significance of differences between the values are illustrated with letters. Different letters indicate significant differences between the experimental values.

ANOVA was applied to the results in order to identify if C/N ratio influenced the production of pigments and biomass in the different conditions tested (Figure 6 and Figure 7). For the multiple comparison test, the REGWQ (Ryan, Einot, Gabriel, Welsh Studentized Range Q test) procedure was conducted under 5% error. The significance of the differences between the mean values were examined and are illustrated with letters. Different letters (in the same color) indicate significant differences between the experimental values.

**Figure 8:**
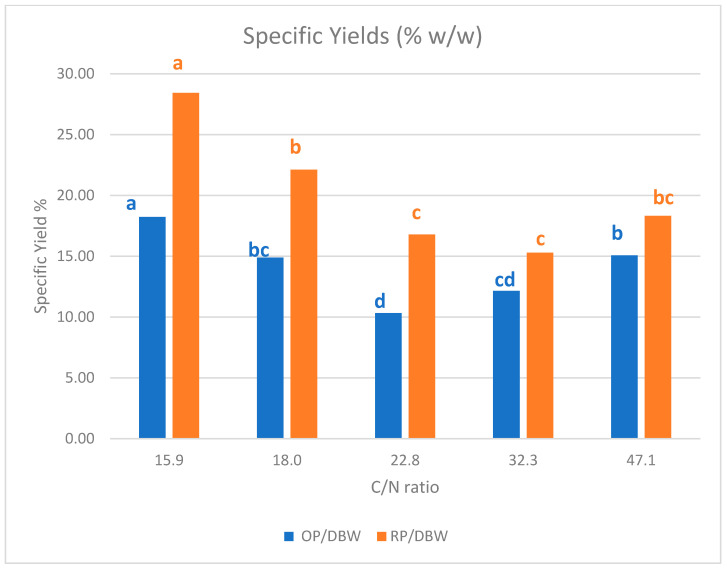
Specific yields for orange pigments (OP) and red pigments (RP) compared to the biomass produced, in relation with the C/N ratio in the culture medium for *T. albobiverticillius* SmF. The significance of differences between the values are illustrated with letters. Different letters (in the same color range) indicate significant differences between the experimental values.

From the ANOVA, a C/N ratio of 22.8 (15 g/L sucrose; 3 g/L yeast extract) induces significantly higher responses in RPY and OPY, compared to all other values (15.9, 18, 32.3, 47.1). This value seems the most appropriate in the chosen range for pigments production. However, this C/N ratio is also the most favorable for biomass production (maximum mean value of 12.84 g/mL). Therefore, the specific production yields, for orange pigment: OP/DBW or for red pigment: RP/DBW (W/W), were calculated to evaluate the efficiency of the production (Figure 8).

In the studied range (15.9 ≤ C/N ≤ 47.1), the C/N ratio shows a clear impact on the red and orange pigments production efficiency (%*w*/*w*) with an optimum impact for the edge C/N values (15.9, 18, and 47.1). Approximately 22.8 and 32.3 the efficiency is lower (Figure 8). This implies that the amounts of pigments produced are higher when the biomass increases but the production efficiency is related to C/N ratio.

### 3.7. Effectiveness of OVAT and CDD on Process Optimization for Pigments and Biomass Production

In our work, a number of variables influencing the fungal growth and pigment production in SmF were studied: five different carbon sources (glucose, sucrose, soluble starch, malt extract fructose), four nitrogen sources (peptone, tryptone, yeast extract, NaNO_3_), and the variations in concentrations for some key parameters: yeast extract (2–4 g/L), KH_2_PO_4_ (0.5–1.5), and MgSO_4_·7H_2_O (0.1–0.3 g/L). The complexity of the interactions between these different factors guided us to combine two statistical approaches (OVAT and CCD) in order to optimize the process.

Considering the minimum and the maximum values experimentally obtained with OVAT method, 8.2, 9.4, and 7.2-fold increase were observed for OPY, RPY, and DBW respectively (Table 7). As for CCD, it allowed, respectively, 1.6, 2, and 3.2-fold increase for the studied range of the parameters. The overall incremental factors (from minimum values to maximum values experimentally obtained in this work) were, therefore, 8.4 fold for OPY, 10 fold for RPY, and 17.4 fold for DBW.

## 4. Discussion

It was extensively shown that media conditions have varying effects on the production of microbial specialized metabolites [81,82]. Moreover, it is well known that secondary metabolite production in microbes, especially in fungi, is largely influenced by carbon and nitrogen sources, in addition to trace elements [72,83,84]. Carbon and nitrogen sources constitute the major cost of the fermentation medium, and several studies on alternate sources reported achieving economical production of red pigments using various agro-products and byproducts [14,15,16,17,18,19]. This requirement necessitated the present study to optimize carbon and nitrogen sources and other growth factors for *T. albobiverticillius* 30548, so that a commercial production process could be evolved.

One-variable-at-a-time analysis using different carbon sources (glucose, sucrose, fructose, soluble starch, and malt extract) presented varying levels of pigment production. From the results, sucrose was the best source for the production of orange and red pigments by *T. albobiverticillius* 30548. Similarly, Chadni et al. (2017) observed a maximum pigment production in *T. verrucosum* if sucrose was used as a carbon source, compared to fructose, glucose, maltose, or starch [85]. However, fructose is the second-best carbon source highlighted in this study, in regards to the production of orange and red pigments. The efficiency of pigment production on fructose was also presented for *T. verrucosum* in Chadni et al. (2017) [85]. Moreover, studies by Tseng et al. (2000) have shown that fructose used as a carbon source showed maximum pigment production in *Monascus purpureus*, while lactose was more convenient for *M. ruber* [86,87].

However, other studies reported that pigment production was more important in the presence of glucose, i.e., in the case of *Monascus* species [65,88,89,90,91,92]. Additionally, there was an increase in biomass, as well as pigment production, with increasing glucose concentration. Some workers also reported that a high glucose concentration (50 g/L) leads to low growth rates and pigment synthesis, indicating an inhibitory action [93]. Similarly, results from Debdulal et al. (2016) suggested that the maximum production of pigments in *Pezicula* sp. BDF 9/1 was obtained with glucose as a carbon source [94].

In other species, Cho et al. (2002) reported maximum pigment production with soluble starch medium from the fungus *Paecilomyces sinclairii* [36]. In a study by Kim et al. (1998) on the bacterial strain *Serratia* sp. KH-95, sucrose gave a maximum yield of yellow pigment [95]. They suggested it may be due to the fact that sucrose can be easily assimilated in the metabolic pathway for biosynthesis of pigment production.

Utilization of different nitrogen sources in the production media had effects not only on fungal growth and pigment production, but also on the biosynthesis of other specialized metabolites [96,97]. Commonly, the pigment structure depends on the form of nitrogen nutrients. For that reason, organic and inorganic nitrogen sources were tested in combination with different carbon sources. Most fungal strains are capable of utilizing organic and inorganic nitrogen, but various research reports show that inorganic nitrogen does not influence pigment formation, but organic nitrogen promotes growth as well as pigmentation [98]. From our experiments, sodium nitrate used as an inorganic source was found unfavorable for growth, as well as pigment production in *T. albobiverticillius* 30548, compared to complex nitrogen sources as peptone, tryptone, or yeast extract. In the literature, the effect of inorganic nitrogen varies depending on the species and strain. For example, nitrate was the better source for producing the pigment PP-V in *Penicillium purpurogenum* IAM15392 cultivation [99]. Comparably, sodium nitrate used as an inorganic nitrate gave intermediate yields, i.e., in the case of *Monascus anka* (M-9) (3 g/L) or *M. kaoliang* KB9 (0.2 g/L). Nevertheless, the highest specific orange pigment production, which is 70.2 mg/g, with an initial pH 6.5, was reached using NaNO_3_, and 68.5 mg/g with an initial pH 2.5 using peptone as the nitrogen source in *Monascus purpureus* [100].

In *Thermomyces sp*., ammonium and peptone serves as a good nitrogen source for pigment production that yielded better growth [101]. Nitrogen under inorganic form seems to have little influence on the formation of pigments, while the organic nitrogen contained in malt extract, yeast extract, peptone, soybean flour or their combination, amplifies fungal growth as well as the production of pigments [102,103]. The study of the impact of different sources of nitrogen on the production of pigments by certain species of *Monascus* demonstrated that, when the nitrogen source is yeast extract or nitrate, red pigments are mainly formed. When ammonium or ammonium nitrate are used, orange pigments are predominant [11,104]. Similarly, when fungal growth is concerned, yeast extract stimulates conidiation, inhibits the sexual cycle, and increases biomass production [105]. This is in agreement with the pigment production and biomass growth for *T. albobiverticillius* 30548 using yeast extract.

The diversity of these results shows that even if the nature of the carbon source is crucial, some other factors may absolutely be cross-analyzed, especially the intrinsic potential of the microbial strain involved, and its enzymatic properties, the nature of the nitrogen source, the C/N ratio, and the presence and concentration of key oligo elements. Indeed, it is well known that if a fungus has favorable nutrient concentrations and appropriate C/N ratio, it will mainly produce biomass representing the fungal growth. However, when an imbalance appears, it will produce pigments expressing as a stress factor for the fungus [106,107,108]. Thus, carbon and nitrogen concentrations as well as C:N ratios have significant impact, which is proven to be strain dependent [109]. As seen in Figure 1 *T. albobiverticillius* 30548 produces high amounts of orange (1.32 g/L quinizarin equivalent), and red pigments (2.16 g/L RYrp equivalent), with the concentrations of 15 g/L sucrose and 3 g/L yeast extract. This medium shows a C/N = 22.8, which is also the ratio where the biomass is maximum (12.84 g/L). However, C/N edge values of 15.9, 18, and 47.1 demonstrate a higher efficiency for the pigment production per gram of dry biomass weight.

The orange pigment yield (OPY) with media optimized using central composite design was found to be 1.43 g/L, which is slightly superior (+2.9%) to the pigments obtained in media optimized by conventional one-variable-at-time method (1.39 g/L). Similarly, for red pigment yield (RPY), it was about 2.59 g/L using response surface analysis, which is 6.15% higher than that obtained using OVAT analysis (2.44 g/L). On the contrary, biomass yield was significantly higher using central composite design experiments (15.98 g/L) compared to OVAT analysis, in which the biomass weight was quite low (6.60 g/L). The difference in biomass increase among the OVAT and CCD methods, may be due to the utilization of additional metal salt (0.25 KCl) in OVAT analysis, and the introduction of KH_2_PO_4_/MgSO_4_·7H_2_O in CDD experiments, which were added to identify the most influencing parameters for fungal growth and pigment production.

Trace elements are considered as a highly important parameter to reach higher growth rates and biomass production. In our study, their different amount did not significantly change the pigment production from the selected fungi. In *T. albobiverticillius* 30548, red pigment production was predicted to be three to four times higher than orange pigment yield and dry biomass weight under the influence of cofactors MgSO_4_·7H_2_O and KH_2_PO_4_ (Figure 4, Figure 5 and Figure 6). A study on *Monascus anka* showed that the mass of dry cells was three to four times higher when trace elements were added in the fed-batch, compared to a conventional culture. At the same time, the total pigment production decreased from 9.0 to 14.6%. It is therefore possible that a high concentration of MgSO_4_ exerted a downregulatory effect on the pigment synthase(s) activity. These results could indicate that the addition of carbon and nitrogen sources would only facilitate cell growth, while trace elements would be key factors, improving cell growth, but also controlling the synthesis of pigments in the high-density culture [70].

However, one of the only trace elements clearly capable of enhancing growth as well as pigment production in *Monascus* species is zinc [110,111,112]. This effect could be due to the involvement of zinc in the absorption and utilization of the carbon source. According to Lin et al. (1991), high concentrations of phosphate and magnesium sulfate (MgSO_4_) inhibit pigment production, with mycelial growth increasing proportionately to MgSO_4_ concentration, in the range of 0.5 to 16 mM [49]. This is not in agreement with the results obtained in the experiments conducted using *T. albobiverticillius* 30548.

The complexity of the interactions between the key factors influencing the fungal growth and pigments productions guided us to combine two statistical approaches (OVAT and CCD) in order to optimize the process. Considering the minimum and the maximum values experimentally obtained, coupling OVAT method and CCD analysis enabled 8.4, 10, and 17.4 incremental factors for OPY, RPY, and DBW, respectively. In our study, the maximal incremental factors were obtained from OVAT as the related experiments focused on the nature of the favorable carbon and nitrogen sources. Indeed, these necessarily have significant impacts on the productions. However, the determination of the most efficient levels (concentrations) of the key factors and their interactions were more easily studied using CCD analysis. This demonstrates that these mathematical tools are complementary and extremely useful in improving the production yields in fungal cultivation, with a minimum of experiments, according to the targeted objectives.

## 5. Conclusions

In the present study, optimization of media components was screened to produce orange and red pigments as well as biomass using *T. albobiverticillius* 30548 by means of one-factor-at-a-time method (OVAT) and central composite design (CCD). The classical one-factor-at-a-time method indicated sucrose, fructose, yeast extract to be high-pigment-yielding media components. Trace elements, such as KH_2_PO_4_ and MgSO_4_·7H_2_O, were other significant factors contributing to pigment production in combination with yeast extract. The CCD design was useful to define the appropriate concentration of media components and to check their interactions. In the cultivation conditions defined with the quadratic model, a threefold increase in biomass production was noted within 148 h of cultivation time. Using the statistical techniques for media optimization, a higher yield of pigment production was also possible, in comparison with the conventional method of optimization. A decrease in cultivation time using a lower concentration of components (yeast extract–2.9 g/L, KH_2_PO_4_–0.9 g/L, and MgSO_4_·7H_2_O–0.2 g/L) is the major outcome of this work. The yield of pigments could also be increased by using fed-batch fermentation, which could overcome the possible problem of substrate inhibition. Simultaneously, this study reflects the wavering nature of *T. albobiverticillius* 30548 towards growth and pigment production using different nutrient sources at different concentrations.

## Figures and Tables

**Table 1:** Experimental range and levels of the three independent variables for the central composite design (CCD) matrix in terms of coded and actual factors.

Variables	Symbol	Coded and Actual Values (in g/L)				
		−α *	−1	0	+1	+α
Yeast extract	X_1_	1.32	2	3	4	4.68
KH_2_PO_4_	X_2_	0.16	0.5	1	1.5	1.84
MgSO_4_·7H_2_O	X_3_	0.03	0.1	0.2	0.3	0.37

* α: axial point.

**Table 2:** Central composite experimental design matrix with observed responses over process parameters (OPY, RPY, DBW) in *T. albobiverticillius* 30548 SmF, using the three independent variables: yeast extract, KH_2_PO_4_, MgSO_4._7H_2_O.

Std Order	Run Order		Variables	(g/L)		Responses	
		X_1_ Yeast Extract	X_2_ KH_2_PO_4_	X_3_ MgSO_4_·7H_2_O	OPY (g/L ^1^)	RPY (g/L ^2^)	DBW ^3^ (g/L)
12	1	3	1.84	0.2	1.13	1.37	10.95
2	2	4	0.5	0.1	1.01	1.52	6.49
3	3	2	1.5	0.1	1.09	1.43	9.03
9	4	1.31	1	0.2	1.04	1.28	7.05
5	5	2	0.5	0.3	1.26	1.47	10.08
20	6	3	1	0.2	1.42	2.59	14.23
13	7	3	1	0.03	1.17	1.91	9.12
4	8	4	1.5	0.1	1.04	1.52	6.81
17	9	3	1	0.2	1.43	2.57	13.63
11	10	3	0.16	0.2	1.24	1.37	10.57
18	11	3	1	0.2	1.42	2.58	13.12
19	12	3	1	0.2	1.43	2.57	14.98
10	13	4.68	1	0.2	0.91	1.43	5.03
1	14	2	0.5	0.1	1.13	1.44	8.89
8	15	4	1.5	0.3	1.09	1.57	8.95
7	16	2	1.5	0.3	1.17	1.51	10.27
15	17	3	1	0.2	1.42	2.57	15.98
16	18	3	1	0.2	1.41	2.58	14.77
6	19	3	1	0.2	1.16	1.57	9.03
14	20	3	1	0.37	1.34	2.03	14.89

^1^: OPY (orange pigment yield at 470 nm in terms of g/L quinizarin equivalent); ^2^: RPY (red pigment yield at 500 nm in terms of g/L red yeast rice pigment equivalent); ^3^: DBW (g/L dry biomass concentration in the culture broth).

**Table 3:** Analysis of variance (ANOVA) for the effect of independent variables on dependent variables and regression coefficients, of the fitted quadratic equations on orange pigment yield (OPY).

Source	DF	Orange Pigment Yield OPY (g/L *)			
		Sum of Squares	Mean Square	F-Value	*p*-Value
Model	9	0.52	0.058	51.09	<0.0001
A-Yeast extract	1	0.024	0.024	208.28	0.0001
B-KH_2_PO_4_	1	9.228 × 10^−3^	9.228 × 10^−3^	81.18	0.0001
C-MgSO_4_·7H_2_O	1	0.035	0.035	311.95	0.0001
AB	1	1.013 × 10^−3^	1.013 × 10^−3^	8.91	0.0137
AC	1	1.250 × 10^−5^	1.250 × 10^−5^	0.11	0.7470
BC	1	2.812 × 10^−3^	2.812 × 10^−3^	24.74	0.0006
A^2^	1	0.36	0.36	3181.99	<0.0001
B^2^	1	0.10	0.10	898.39	<0.0001
C^2^	1	0.051	0.051	447.79	<0.0001
Residual	10	1.137 × 10^−3^	1.137 × 10^−4^		
Lack of Fit	5	8.534 × 10^−4^	1.707 × 10^−4^	3.01	0.1258
Pure Error	5	2.833 × 10^−4^	5.667 × 10^−5^		
Cor. Total	19	0.52			
SD		0.001			
Mean		1.22			
CV%		0.88			
R^2^		0.9978			
R^2^_adj._		0.9959			
R^2^_pred._		0.9859			
Adeq. Pre.		68.726			

*: in g/L quinizarin equivalent; R^2^: determination coefficient; R^2^_adj_: adjusted R^2^; R^2^_pred:_ predicted R^2^; CV %: coefficient of variation; Adeq. Pre: predicted adequacy; *p* < 0.01 highly significant; 0.01< *p* <0.05 significant; *p* > 0.05 not significant.

**Table 4:** Analysis of variance (ANOVA) for the effect of independent variables on dependent variables and regression coefficients, of the fitted quadratic equations on red pigment yield (RPY).

Source	DF	Red Pigment Yield RPY (g/L *)			
		Sum of Squares	Mean Square	F-Value	*p*-Value
Model	9	5.14	0.57	55.81	<0.0001
A-Yeast extract	1	0.025	0.025	242.44	<0.0001
B-KH_2_PO_4_	1	6.590 × 10^−5^	6.590 × 10^−5^	0.64	0.04411
C-MgSO_4_·7H_2_O	1	0.012	0.012	121.27	<0.0001
AB	1	1.125 × 10^−4^	1.125 × 10^−4^	1.10	0.3192
AC	1	1.250 × 10^−5^	1.250 × 10^−5^	0.12	0.7340
BC	1	3.125 × 10^−4^	3.125 × 10^−4^	3.05	0.1112
A^2^	1	2.69	2.69	26252.50	<0.0001
B^2^	1	2.62	2.62	25611.75	<0.0001
C^2^	1	0.66	0.66	6473.14	<0.0001
Residual	10	1.024 × 10^−3^	1.024 × 10^−4^		
Lack of Fit	5	6.907 × 10^−4^	1.381 × 10^−4^	2.07	0.2215
Pure Error	5	3.333 × 10^−4^	6.667 × 10^−5^		
Cor. Total	19	5.15			
SD		0.001			
Mean		1.84			
CV%		0.55			
R^2^		0.9998			
R^2^_adj._		0.9996			
R^2^_pred._		0.9988			
Adeq. Pre.		180.745			

*: in g/L Red Yeast rice pigments (RYrp) equivalent R^2^: determination coefficient; R^2^_adj_: adjusted R^2^; R^2^_pred:_ predicted R^2^; CV%: coefficient of variation; Adeq. Pre: predicted adequacy; *p* < 0.01 highly significant; 0.01 < *p* < 0.05 significant; *p* > 0.05 not significant.

**Table 5:** Analysis of variance (ANOVA) for the effect of independent variables on dependent variables and regression coefficients, of the fitted quadratic equations on dry biomass concentration (DBW).

Source	DF	Dry Biomass Concentration (DBW g/L)			
		Sum of Squares	Mean Square	F-Value	*p*-Value
Model	9	191.65	21.29	23.77	<0.0001
A-Yeast extract	1	7.90	7.90	8.82	0.0141
B-KH_2_PO_4_	1	0.11	0.11	0.12	0.7368
C-MgSO_4_·7H_2_O	1	20.70	20.70	23.10	0.0007
AB	1	1.013 × 10^−3^	1.013 × 10^−3^	1.130 × 10^−3^	0.9738
AC	1	0.63	0.63	0.71	0.4203
BC	1	0.015	0.015	0.017	0.8986
A^2^	1	138.80	138.80	154.92	<0.0001
B^2^	1	29.66	29.66	33.11	0.0002
C^2^	1	14.25	14.25	15.91	0.0026
Residual	10	8.96	0.90		
Lack of Fit	5	3.75	0.75	0.72	0.6372
Pure Error	5	5.21	1.04		
Cor. Total	19	200.61			
SD		0.95			
Mean		10.69			
CV%		8.85			
R^2^		0.9553			
R^2^_adj._		0.9151			
R^2^_pred._		0.8209			
Adeq. Pre.		15.026			

R^2^: Determination coefficient; R^2^_adj_: adjusted R^2^; R^2^_pred.:_ predicted R^2^; CV%: coefficient of variation; Adeq. Pre: predicted adequacy; *p* < 0.01 highly significant; 0.01 < *p* < 0.05 significant; *p* > 0.05 not significant.

**Table 6:** C/N ratio on OPY, RPY, and DBW in *T. albobiverticillius* 30548 SmF in central composite design (CCD) experiments.

C/N	n	Mean OPY ^1^ g/L	SD	Mean RPY ^2^ g/L	SD	Mean DBW ^3^ g/L	SD
15.9	3	0.92	0.04	1.43	0.03	5.03	0.06
18	12	1.01	0.08	1.51	0.07	6.81	1.64
22.8	33	1.32	0.12	2.16	0.52	12.84	2.38
32.3	12	1.16	0.07	1.46	0.04	9.57	0.71
47.1	3	1.07	0.03	1.30	0.01	7.08	0.03

^1^: OPY (orange pigment yield at 470 nm in terms of g/L quinizarin equivalent); ^2^: RPY (red pigment yield at 500 nm in terms of g/L red yeast rice pigment equivalent); ^3^: DBW (g/L dry biomass concentration in the culture broth).

**Table 7:** Effectiveness of OVAT (one-variable-at-a-time) and CCD (central composite design) on process optimization for orange and red pigments yields (OPY and RPY) and biomass (DBW) production in SmF for *T. albobiverticillius* 30548.

**C and N Sources**	**Min. Values g/L**			**Max. Values g/L**			**Incremental Factors**		
	**OPY**	**RPY**	**DBW**	**OPY**	**RPY**	**DBW**	**OPY**	**RPY**	**DBW**
***2 Factors Combination***									
	ME */Peptone	Sucrose/NaNO_3_	ME */NaNO_3_	Sucrose/YE **	Sucrose/YE **	Sucrose/YE **			
OVAT optimization	0.17	0.26	0.92	1.39	2.44	6.60	×8.2	×9.4	×7.2
***4 Factors Concentrations*** (Sucrose/YE **^/^KH_2_PO_4_/MgSO_4_·7H_2_O)									
CCD optimization	0.91	1.28	5.03	1.43	2.,59	15.98	×1.6	×2	×3.2
**Overall increase**							×8.4	×10	×17.4

* ME: Malt extract ** YE: Yeast Extract.

## Data Availability

The data presented in this study are available on request from the corresponding author.
